# Ethyl 3-[2-(*p*-tolyl­carbamothio­yl)hydrazinyl­idene]butano­ate

**DOI:** 10.1107/S160053681004290X

**Published:** 2010-10-30

**Authors:** Yan-Ling Zhang, Xu-Feng Hou, Fu-Juan Zhang, Feng-Ling Yang

**Affiliations:** a College of Chemistry and Chemical Engineering, Xuchang University, Henan 461000, People’s Republic of China

## Abstract

The title compound, C_14_H_19_N_3_O_2_S, was obtained from a condensation reaction of *N*-(*p*-tol­yl)hydrazinecarbothio­amide and ethyl acetoacetate. The mol­ecule assumes an *E* configuration; the thio­semicarbazide and ester groups are located on the opposite sides of the C=N bond. The almost planar thio­semicarbazide unit (r.m.s. deviation = 0.0130 Å) is tilted at a dihedral angle of 49.54 (12)° with respect to the benzene ring. Inter­molecular N—H⋯N and N—H⋯S hydrogen bonding stabilizes the crystal structure. The eth­oxy group of the ester unit is disordered over two positions, with a site-occupancy ratio of 0.680 (10):0.320 (10).

## Related literature

For biological applications of thio­semicarbazones, see: Okabe *et al.* (1993 ▶); Hu *et al.* (2006 ▶). For related structures, see: Zhang *et al.* (2005 ▶); Shan & Zhang (2006 ▶).
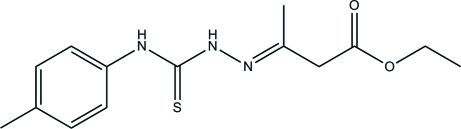


## Experimental

### 

#### Crystal data

C_14_H_19_N_3_O_2_S
*M* *_r_* = 293.38
Orthorhombic, 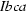

*a* = 14.1747 (3) Å
*b* = 25.1439 (4) Å
*c* = 17.4381 (2) Å
*V* = 6215.08 (17) Å^3^
*Z* = 16
Cu *K*α radiation
μ = 1.90 mm^−1^
*T* = 293 K
0.20 × 0.18 × 0.18 mm

#### Data collection

Oxford Diffraction Xcalibur Eos Gemini diffractometer
Absorption correction: multi-scan (*CrysAlis PRO*; Oxford Diffraction, 2009 ▶) *T*_min_ = 0.703, *T*_max_ = 0.726
6852 measured reflections
2775 independent reflections
2380 reflections with *I* > 2σ(*I*)
*R*_int_ = 0.018

#### Refinement

*R*[*F* ^2^ > 2σ(*F* ^2^)] = 0.057
*wR*(*F* ^2^) = 0.175
*S* = 1.07
2775 reflections
199 parameters
H atoms treated by a mixture of independent and constrained refinement
Δρ_max_ = 0.33 e Å^−3^
Δρ_min_ = −0.37 e Å^−3^

### 

Data collection: *CrysAlis CCD* (Oxford Diffraction, 2008 ▶); cell refinement: *CrysAlis RED* (Oxford Diffraction, 2008 ▶); data reduction: *CrysAlis RED*; program(s) used to solve structure: *SHELXTL* (Sheldrick, 2008 ▶); program(s) used to refine structure: *SHELXTL*; molecular graphics: *SHELXTL*; software used to prepare material for publication: *SHELXTL*.

## Supplementary Material

Crystal structure: contains datablocks I, global. DOI: 10.1107/S160053681004290X/xu5056sup1.cif

Structure factors: contains datablocks I. DOI: 10.1107/S160053681004290X/xu5056Isup2.hkl

Additional supplementary materials:  crystallographic information; 3D view; checkCIF report

## Figures and Tables

**Table 1:** Hydrogen-bond geometry (Å, °)

*D*—H⋯*A*	*D*—H	H⋯*A*	*D*⋯*A*	*D*—H⋯*A*
N1—H1*N*⋯N3^i^	0.81 (3)	2.54 (3)	3.300 (3)	155 (2)
N2—H2*B*⋯S1^ii^	0.86	2.85	3.5572 (18)	141

Symmetry codes: (i) 
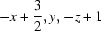
; (ii) 
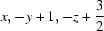
.

**Fig. 1.**
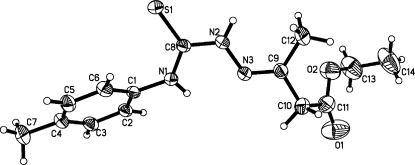
The molecular structure of the title compound, with displacement ellipsoids drawn at 30% probability level.

**Fig. 2.**
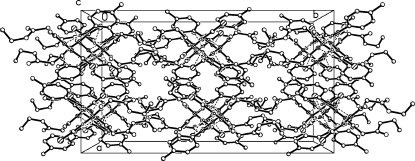
The packing diagram of the title compound. Intermolecular hydrogen bonds are shown as dashed line.

## supplementary crystallographic information

### Comment

Thiosemicarbazones have attracted much attention as they show potential application in the biological field (Okabe *et al.*, 1993; Hu *et al.*, 2006). There are a few single-crystal reports about them (Zhang *et al.*, 2005; Shan *et al.*, 2006). Detailed information on their molecular and crystal structures is necessary to understand their anticancer activity. The molecular structure of (I) is shown in Fig 1. The molecule of (I) exhibits an E configuration. The thiosemicarbazide and Ethyl acetoacetate unit are located on opposite sides of the N3=C9 bond. The thiosemicarbazide unit has a planar configuration and is tilted with respect to the *p*-methylphenyl mean plane, forming a dihedral angle of 49.54 (12)°.In the crystal structure of the title compound, there are N(1)—H(1 N)···N(3)#1, N(1)—H(1 N)···N(1)#1 and N(2)—H(2B)···S(1)#2 hydrogen-bond interactions in molecules (Fig. 2).

### Experimental

*N*-(*p*-Tolyl)thiosemicarbazide (1.8 g,10 mmol) and ethyl acetoacetate (1.3 g, 10 mmol) was dissolved in 95% ethanol (15 ml) and the solution was refluxed for 2 h. Fine colorless crystals appeared on cooling. They were filtered and washed by 95% ethanol to give 2.13 g of the title compound in 71.7% yield. Single crystals suitable for X-ray measurements were obtained from mother liquid by slow evaporation at room temperature.

### Refinement

The H1N atom was located in a difference Fourier map and refined isotropically. Other H atoms were placed in calculated positions with C—H = 0.93-0.97 and N—H = 0.86 Å, and refined using a riding model, *U*_iso_(H) = 1.5U_eq_(C) for methyl H atoms and 1.2U_eq_(C,N) for the others. The ethoxy part of the ester unit is disordered over two positions with site occupancies ratio of 0.680 (10):0.320 (10).

### Crystal data

**Table:**

C_14_H_19_N_3_O_2_S	*F*(000) = 2496
*M**_r_* = 293.38	*D*_x_ = 1.254 Mg m^−^^3^
Orthorhombic, *I**b**c**a*	Cu *K*α radiation, λ = 1.54184 Å
Hall symbol: -I 2b 2c	Cell parameters from 4042 reflections
*a* = 14.1747 (3) Å	θ = 3.1–72.2°
*b* = 25.1439 (4) Å	µ = 1.90 mm^−^^1^
*c* = 17.4381 (2) Å	*T* = 293 K
*V* = 6215.08 (17) Å^3^	Prismatic, colorless
*Z* = 16	0.20 × 0.18 × 0.18 mm

### Data collection

**Table:**

Oxford Diffraction Xcalibur Eos Gemini diffractometer	2775 independent reflections
Radiation source: fine-focus sealed tube	2380 reflections with *I* > 2σ(*I*)
graphite	*R*_int_ = 0.018
ω scans	θ_max_ = 67.0°, θ_min_ = 3.5°
Absorption correction: multi-scan (*CrysAlis PRO*; Oxford Diffraction, 2009)	*h* = −16→13
*T*_min_ = 0.703, *T*_max_ = 0.726	*k* = −30→29
6852 measured reflections	*l* = −20→10

### Refinement

**Table:**

Refinement on *F*^2^	Primary atom site location: structure-invariant direct methods
Least-squares matrix: full	Secondary atom site location: difference Fourier map
*R*[*F*^2^ > 2σ(*F*^2^)] = 0.057	Hydrogen site location: inferred from neighbouring sites
*wR*(*F*^2^) = 0.175	H atoms treated by a mixture of independent and constrained refinement
*S* = 1.07	*w* = 1/[σ^2^(*F*_o_^2^) + (0.111*P*)^2^ + 3.488*P*] where *P* = (*F*_o_^2^ + 2*F*_c_^2^)/3
2775 reflections	(Δ/σ)_max_ = 0.001
199 parameters	Δρ_max_ = 0.33 e Å^−^^3^
0 restraints	Δρ_min_ = −0.36 e Å^−^^3^

### Special details

**Table:**

Geometry. All e.s.d.'s (except the e.s.d. in the dihedral angle between two l.s. planes) are estimated using the full covariance matrix. The cell e.s.d.'s are taken into account individually in the estimation of e.s.d.'s in distances, angles and torsion angles; correlations between e.s.d.'s in cell parameters are only used when they are defined by crystal symmetry. An approximate (isotropic) treatment of cell e.s.d.'s is used for estimating e.s.d.'s involving l.s. planes.
Refinement. Refinement of *F*^2^ against ALL reflections. The weighted *R*-factor *wR* and goodness of fit *S* are based on *F*^2^, conventional *R*-factors *R* are based on *F*, with *F* set to zero for negative *F*^2^. The threshold expression of *F*^2^ > σ(*F*^2^) is used only for calculating *R*-factors(gt) *etc*. and is not relevant to the choice of reflections for refinement. *R*-factors based on *F*^2^ are statistically about twice as large as those based on *F*, and *R*- factors based on ALL data will be even larger.

### Fractional atomic coordinates and isotropic or equivalent isotropic displacement parameters (Å^2^)

**Table:**

	*x*	*y*	*z*	*U*_iso_*/*U*_eq_	Occ. (<1)
S1	0.89584 (5)	0.56086 (3)	0.67758 (3)	0.0567 (3)	
N1	0.84677 (12)	0.51980 (8)	0.54081 (10)	0.0426 (4)	
N2	0.78883 (13)	0.47864 (8)	0.64691 (10)	0.0468 (5)	
H2B	0.7853	0.4742	0.6957	0.056*	
N3	0.74049 (14)	0.44509 (7)	0.59763 (10)	0.0456 (5)	
C1	0.88957 (13)	0.55867 (8)	0.49298 (12)	0.0405 (5)	
C2	0.93995 (16)	0.54086 (9)	0.42997 (11)	0.0449 (5)	
H2A	0.9461	0.5046	0.4210	0.054*	
C3	0.98110 (17)	0.57697 (10)	0.38031 (13)	0.0521 (6)	
H3A	1.0146	0.5646	0.3381	0.063*	
C4	0.97320 (18)	0.63105 (10)	0.39238 (14)	0.0569 (6)	
C5	0.9206 (2)	0.64823 (10)	0.45486 (15)	0.0595 (6)	
H5A	0.9137	0.6845	0.4636	0.071*	
C6	0.87831 (18)	0.61255 (10)	0.50432 (14)	0.0513 (5)	
H6A	0.8423	0.6249	0.5452	0.062*	
C7	1.0220 (3)	0.66988 (14)	0.33947 (19)	0.0859 (10)	
H7A	1.0114	0.7055	0.3573	0.129*	
H7B	0.9970	0.6662	0.2886	0.129*	
H7C	1.0885	0.6627	0.3389	0.129*	
C8	0.84189 (14)	0.51858 (9)	0.61720 (11)	0.0420 (5)	
C9	0.68544 (16)	0.41050 (9)	0.62652 (13)	0.0479 (5)	
C10	0.6300 (2)	0.37820 (11)	0.56998 (17)	0.0627 (7)	
H10A	0.6424	0.3924	0.5193	0.075*	
H10B	0.5636	0.3838	0.5805	0.075*	
C11	0.6470 (3)	0.31894 (13)	0.56713 (19)	0.0735 (8)	
C12	0.6676 (2)	0.40351 (14)	0.71046 (17)	0.0765 (9)	
H12A	0.7242	0.3913	0.7350	0.115*	
H12B	0.6183	0.3778	0.7178	0.115*	
H12C	0.6489	0.4369	0.7324	0.115*	
O1	0.6285 (3)	0.29160 (13)	0.5138 (2)	0.1299 (13)	
C13	0.7213 (7)	0.2436 (2)	0.6246 (4)	0.113 (3)	0.680 (10)
H13A	0.7212	0.2299	0.5726	0.135*	0.680 (10)
H13B	0.7831	0.2372	0.6466	0.135*	0.680 (10)
C14	0.6501 (10)	0.2163 (3)	0.6696 (5)	0.143 (4)	0.680 (10)
H14A	0.6632	0.1789	0.6701	0.214*	0.680 (10)
H14B	0.5891	0.2224	0.6473	0.214*	0.680 (10)
H14C	0.6509	0.2297	0.7211	0.214*	0.680 (10)
O2	0.7016 (4)	0.30089 (13)	0.6237 (2)	0.0891 (18)	0.680 (10)
C13'	0.6327 (18)	0.2371 (11)	0.6442 (15)	0.138 (8)*	0.320 (10)
H13C	0.6033	0.2200	0.6005	0.165*	0.320 (10)
H13D	0.5973	0.2272	0.6896	0.165*	0.320 (10)
C14'	0.7322 (16)	0.2172 (10)	0.6520 (14)	0.137 (7)*	0.320 (10)
H14D	0.7318	0.1792	0.6561	0.206*	0.320 (10)
H14E	0.7603	0.2322	0.6972	0.206*	0.320 (10)
H14F	0.7680	0.2276	0.6078	0.206*	0.320 (10)
O2'	0.6275 (10)	0.2960 (5)	0.6341 (7)	0.115 (4)*	0.320 (10)
H1N	0.8246 (18)	0.4946 (12)	0.5181 (15)	0.046 (7)*	

### Atomic displacement parameters (Å^2^)

**Table:**

	*U*^11^	*U*^22^	*U*^33^	*U*^12^	*U*^13^	*U*^23^
S1	0.0723 (5)	0.0606 (4)	0.0371 (4)	−0.0171 (3)	−0.0054 (2)	−0.0039 (2)
N1	0.0470 (9)	0.0466 (10)	0.0342 (9)	−0.0067 (8)	−0.0016 (7)	−0.0005 (7)
N2	0.0538 (10)	0.0543 (11)	0.0322 (8)	−0.0077 (8)	0.0016 (7)	0.0029 (7)
N3	0.0515 (10)	0.0467 (10)	0.0386 (9)	−0.0045 (8)	−0.0002 (8)	0.0017 (7)
C1	0.0393 (10)	0.0482 (11)	0.0341 (10)	−0.0022 (8)	−0.0036 (7)	0.0046 (8)
C2	0.0517 (11)	0.0466 (11)	0.0365 (10)	−0.0001 (9)	0.0001 (9)	0.0009 (8)
C3	0.0532 (12)	0.0639 (14)	0.0392 (11)	−0.0003 (11)	0.0042 (9)	0.0060 (10)
C4	0.0628 (14)	0.0594 (14)	0.0486 (12)	−0.0090 (12)	−0.0029 (11)	0.0148 (10)
C5	0.0755 (15)	0.0432 (12)	0.0599 (14)	0.0016 (11)	−0.0050 (12)	0.0069 (10)
C6	0.0582 (12)	0.0512 (12)	0.0447 (11)	0.0072 (10)	0.0020 (10)	0.0012 (10)
C7	0.105 (3)	0.080 (2)	0.0724 (18)	−0.0259 (19)	0.0066 (18)	0.0257 (16)
C8	0.0431 (10)	0.0485 (11)	0.0343 (10)	0.0003 (8)	−0.0005 (8)	0.0004 (8)
C9	0.0478 (11)	0.0475 (11)	0.0483 (12)	−0.0010 (9)	0.0000 (9)	0.0068 (9)
C10	0.0680 (15)	0.0528 (14)	0.0674 (16)	−0.0119 (12)	−0.0098 (13)	0.0079 (12)
C11	0.087 (2)	0.0642 (17)	0.0695 (17)	−0.0118 (15)	−0.0066 (15)	−0.0055 (14)
C12	0.0829 (19)	0.090 (2)	0.0560 (15)	−0.0268 (17)	0.0156 (14)	0.0096 (14)
O1	0.172 (3)	0.098 (2)	0.120 (2)	0.027 (2)	−0.048 (2)	−0.0389 (19)
C13	0.166 (7)	0.061 (3)	0.111 (5)	0.016 (4)	0.012 (5)	0.013 (3)
C14	0.250 (13)	0.073 (4)	0.106 (5)	−0.010 (6)	0.047 (7)	0.013 (4)
O2	0.130 (4)	0.0540 (18)	0.083 (2)	0.0084 (19)	−0.021 (2)	0.0044 (15)

### Geometric parameters (Å, °)

**Table:**

S1—C8	1.680 (2)	C10—C11	1.510 (4)
N1—C8	1.334 (3)	C10—H10A	0.9700
N1—C1	1.421 (3)	C10—H10B	0.9700
N1—H1N	0.81 (3)	C11—O1	1.186 (4)
N2—C8	1.357 (3)	C11—O2'	1.332 (12)
N2—N3	1.386 (3)	C11—O2	1.333 (5)
N2—H2B	0.8600	C12—H12A	0.9600
N3—C9	1.272 (3)	C12—H12B	0.9600
C1—C6	1.378 (3)	C12—H12C	0.9600
C1—C2	1.385 (3)	C13—C14	1.450 (13)
C2—C3	1.384 (3)	C13—O2	1.468 (7)
C2—H2A	0.9300	C13—H13A	0.9700
C3—C4	1.381 (4)	C13—H13B	0.9700
C3—H3A	0.9300	C14—H14A	0.9600
C4—C5	1.389 (4)	C14—H14B	0.9600
C4—C7	1.511 (3)	C14—H14C	0.9600
C5—C6	1.381 (4)	C13'—O2'	1.49 (3)
C5—H5A	0.9300	C13'—C14'	1.50 (4)
C6—H6A	0.9300	C13'—H13C	0.9700
C7—H7A	0.9600	C13'—H13D	0.9700
C7—H7B	0.9600	C14'—H14D	0.9600
C7—H7C	0.9600	C14'—H14E	0.9600
C9—C12	1.496 (3)	C14'—H14F	0.9600
C9—C10	1.500 (4)		
			
C8—N1—C1	128.61 (19)	C11—C10—H10B	107.8
C8—N1—H1N	116.7 (18)	H10A—C10—H10B	107.1
C1—N1—H1N	114.7 (18)	O1—C11—O2'	113.0 (6)
C8—N2—N3	119.19 (16)	O1—C11—O2	120.8 (4)
C8—N2—H2B	120.4	O2'—C11—O2	47.5 (6)
N3—N2—H2B	120.4	O1—C11—C10	124.2 (3)
C9—N3—N2	118.29 (19)	O2'—C11—C10	111.4 (6)
C6—C1—C2	119.4 (2)	O2—C11—C10	113.8 (3)
C6—C1—N1	122.8 (2)	C9—C12—H12A	109.5
C2—C1—N1	117.6 (2)	C9—C12—H12B	109.5
C3—C2—C1	120.1 (2)	H12A—C12—H12B	109.5
C3—C2—H2A	119.9	C9—C12—H12C	109.5
C1—C2—H2A	119.9	H12A—C12—H12C	109.5
C4—C3—C2	121.1 (2)	H12B—C12—H12C	109.5
C4—C3—H3A	119.4	C14—C13—O2	109.7 (8)
C2—C3—H3A	119.4	C14—C13—H13A	109.7
C3—C4—C5	118.0 (2)	O2—C13—H13A	109.7
C3—C4—C7	120.4 (3)	C14—C13—H13B	109.7
C5—C4—C7	121.6 (3)	O2—C13—H13B	109.7
C6—C5—C4	121.4 (2)	H13A—C13—H13B	108.2
C6—C5—H5A	119.3	C13—C14—H14A	109.5
C4—C5—H5A	119.3	C13—C14—H14B	109.5
C1—C6—C5	119.9 (2)	H14A—C14—H14B	109.5
C1—C6—H6A	120.0	C13—C14—H14C	109.5
C5—C6—H6A	120.0	H14A—C14—H14C	109.5
C4—C7—H7A	109.5	H14B—C14—H14C	109.5
C4—C7—H7B	109.5	C11—O2—C13	116.9 (5)
H7A—C7—H7B	109.5	O2'—C13'—C14'	113 (2)
C4—C7—H7C	109.5	O2'—C13'—H13C	109.0
H7A—C7—H7C	109.5	C14'—C13'—H13C	109.0
H7B—C7—H7C	109.5	O2'—C13'—H13D	109.0
N1—C8—N2	115.26 (19)	C14'—C13'—H13D	109.0
N1—C8—S1	125.98 (17)	H13C—C13'—H13D	107.8
N2—C8—S1	118.75 (15)	C13'—C14'—H14D	109.5
N3—C9—C12	124.8 (2)	C13'—C14'—H14E	109.5
N3—C9—C10	115.5 (2)	H14D—C14'—H14E	109.5
C12—C9—C10	119.4 (2)	C13'—C14'—H14F	109.5
C9—C10—C11	118.2 (2)	H14D—C14'—H14F	109.5
C9—C10—H10A	107.8	H14E—C14'—H14F	109.5
C11—C10—H10A	107.8	C11—O2'—C13'	121.6 (14)
C9—C10—H10B	107.8		
			
C8—N2—N3—C9	−174.7 (2)	N3—N2—C8—S1	176.99 (15)
C8—N1—C1—C6	−44.9 (3)	N2—N3—C9—C12	0.8 (4)
C8—N1—C1—C2	138.9 (2)	N2—N3—C9—C10	175.7 (2)
C6—C1—C2—C3	2.1 (3)	N3—C9—C10—C11	116.9 (3)
N1—C1—C2—C3	178.3 (2)	C12—C9—C10—C11	−67.9 (4)
C1—C2—C3—C4	0.2 (3)	C9—C10—C11—O1	−158.9 (4)
C2—C3—C4—C5	−1.6 (4)	C9—C10—C11—O2'	60.4 (7)
C2—C3—C4—C7	177.4 (2)	C9—C10—C11—O2	8.7 (5)
C3—C4—C5—C6	0.9 (4)	O1—C11—O2—C13	−11.8 (7)
C7—C4—C5—C6	−178.2 (3)	O2'—C11—O2—C13	82.2 (9)
C2—C1—C6—C5	−2.8 (3)	C10—C11—O2—C13	−180.0 (5)
N1—C1—C6—C5	−178.9 (2)	C14—C13—O2—C11	−90.3 (9)
C4—C5—C6—C1	1.3 (4)	O1—C11—O2'—C13'	30.6 (18)
C1—N1—C8—N2	174.30 (19)	O2—C11—O2'—C13'	−80.9 (16)
C1—N1—C8—S1	−6.5 (3)	C10—C11—O2'—C13'	175.9 (14)
N3—N2—C8—N1	−3.8 (3)	C14'—C13'—O2'—C11	77 (2)

### Hydrogen-bond geometry (Å, °)

**Table:**

*D*—H···*A*	*D*—H	H···*A*	*D*···*A*	*D*—H···*A*
N1—H1N···N3^i^	0.81 (3)	2.54 (3)	3.300 (3)	155 (2)
N2—H2B···S1^ii^	0.86	2.85	3.5572 (18)	141

Symmetry codes: (i) −*x*+3/2, *y*, −*z*+1; (ii) *x*, −*y*+1, −*z*+3/2.
